# Validity Assessment of Referral Decisions at a VA Health Care System Polytrauma System of Care

**DOI:** 10.7759/cureus.240

**Published:** 2015-01-07

**Authors:** Joyce Chung, Fatima Aguila, Odette Harris

**Affiliations:** 1 Department of Veterans Affairs, VA Palo Alto Health Care System; 2 Social Work, VA Palo Alto Health Care System; 3 Department of Neurosurgery, Stanford University School of Medicine

**Keywords:** decision-making, polytrauma, traumatic brain injury, referrals, rehabilitation, veterans, military, admissions, rehabilitation needs, resources

## Abstract

There has been intensive interest to ensure equitable and appropriate access to the specialized rehabilitative services of the VA Polytrauma System of Care (PSC) for patients sustaining polytrauma and traumatic brain injuries (TBI). A retrospective cohort study with prospective data acquisition was conducted to assess validity and objectivity of the acceptance decision algorithm to the VA Palo Alto Health Care System (VAPAHCS) PSC. Our hypotheses are (1) VAPAHCS PSC referral decisions were appropriate and without bias and (2) the identified needs of redirected referrals were addressed. This analysis included 1,025 referrals (906 patients); 813 patients (89.7%) were accepted, and 93 (10.3%) were redirected. Redirected cases were older, were more often active duty service members, and were not from the West Coast. There were more females redirected due to concomitant spinal cord injury. These are rationale differences. In redirected patients, the most commonly identified rehabilitation needs were psychological support, mobility/physical therapy, and communication/speech services; >75% of patients had these services offered elsewhere outside of the PSC resources. While balancing financial stewardship and meeting our mission to provide outstanding rehabilitative care to veterans and service members, we demonstrated that acceptance decisions were valid and without bias, and redirected patients received appropriate alternate resources.

## Introduction

During the recent military conflicts of Operation Iraqi Freedom (OIF) and Operation Enduring Freedom (OEF), blast injuries from widely used improvised explosive devices (IED), often produced injuries to multiple organ systems. This injury pattern has been termed polytrauma. As defined, polytrauma frequently includes a TBI, co-occuring with systemic injuries, such as amputations, orthopedic injuries, vision/hearing impairments, as well as post-traumatic stress disorder (PTSD), and other mental health conditions. Of those injured in recent conflicts, TBI has been among the most frequently occurring injury, with 28-30% of the troops estimated to have suffered an associated TBI. Thus, TBI has emerged as the “signature injury” of this conflict [1-3]. Current statistics estimate that since 2000, nearly 250,000 TBI have been sustained primarily during the OEF/OIF conflict [4].

Recognition of an increased incidence of polytrauma/TBI led the Veterans Affairs Health Care System (VA) to formally establish TBI Lead Centers in collaboration with the Defense and Veterans Brain Injury Center (DVBIC). Building on the accepted model in health care of expertise centralization and demonstration of better outcomes in volume-based centers, these TBI-focused centers optimized health care delivery by responding to the changing demographics and characteristics of these injured service members, including treating the newly recognized polytrauma/TBI condition. These VA TBI Lead Centers received designations as Polytrauma Rehabilitation Centers (PRC) in 2005 as part of the Polytrauma System of Care (PSC) lifetime treatment continuum [5-6]. These centers responded to this emerging unique cohort and provided the necessary cadre of highly specialized clinicians in order to address the often co-occurring conditions involving TBI and other significant systemic injuries. 

The specialization and focus of these centers has led to an intensive interest in ensuring the equitable and appropriate access to the specialized and comprehensive services that the PSC offers to the polytrauma/TBI patient. Efforts, such as using geographic information systems, have aided in addressing these concerns. Nonetheless, the PSC admissions process is challenged not only with addressing the complexity and severity of polytrauma/TBI patients and their associated co-morbidities, including the increased burden of mental health needs and the continual flow of patients, but the admissions process also must keep in focus the stewardship of federal resources.

Given these issues, challenges, and focus on equitable access, we initiated a review of the decision-making process of referrals accepted into the PSC program at the VA Palo Alto Health Care System (VAPAHCS), one of the five PSCs nationwide. We assessed the validity and effectiveness of the decision-making process for all patients referred to the PSC, regardless of their admission status.

Our goal was to better understand the effect of our decisions with specific reference to equitable access within our nation’s designated systems. Additionally, we aimed to query our algorithm, to ensure transparency and validity, and to determine whether the needs of the population as a whole are being appropriately addressed. As the system in place adequately addresses the needs of those admitted, the cohort of those redirected were of equal focus. Thus, concomitantly, we assessed the needs of the redirected referrals and examined whether the received services were adequate to address their identified needs.

Our hypotheses: (1) the VAPAHCS PSC referral decisions were appropriate and without bias, best serving the identified patients’ needs, and (2) the identified needs of all referrals were addressed whether within the PSC, if accepted, or elsewhere, if redirected.

## Materials and methods

### Retrospective cohort analysis: Comparison between accepted and redirected referrals

A retrospective cohort analysis with prospective data acquisition was conducted with a cohort of referrals received at the VAPAHCS PSC. The decision-making team may “accept” the referral into the PSC program under the appropriate admissions criteria, or may “redirect” the referral to another resource that may better meet the identified needs of the patient. The decision-making criteria for acceptance to the PSC assumes that the patients’ needs will be identified and be addressed through comprehensive and appropriate services offered whether through the PSC or through other services outside of the PSC resources. This analysis was part of an ongoing program evaluation initiative and consent was waived. 

Demographic and general diagnostic information on each patient were collected at the time of the referral. Demographic comparisons were made between the accepted referrals and redirected referrals. The patient data was compiled from the VA Computerized Patient Record System (CPRS), the VA electronic medical record system, and if necessary, from referral sources. The CPRS captures all services referred, offered, and received through any VA agency nationwide.

### Cohort compilation

The cohort compiled for this analysis included: all VAPAHCS PSC patient referrals for three consecutive fiscal years (2010-2011). Two quarters of each year (3rd/4th quarters for FY2009, 3rd/4th quarters for FY2010, 1st/2nd quarters for FY2011) were selected for review. The FY2011 quarters were the most recent data available at the time of analysis.

### Identification of redirected referral needs and determination of services received

A data collection instrument was developed with guidance by clinical rehabilitation providers and medical literature [7-8], to collate and assess information on the referral needs of the redirected patients, and to determine whether services were offered and received by the patients to address their identified needs outside of admission to the VAPAHCS PSC. Data from CPRS were collected related to the critical domains of rehabilitation needs, such as mobility/physical therapy, speech language pathology, occupational therapy, medication, sleep hygiene, nutrition/feeding, psychological support, vision, hearing, and pain management. The data collection instrument solicited information on TBI status and severity, co-morbidities, referral source, specific rehabilitation, and health/wellness needs. Data collection included review of whether the patient was offered and received services to meet identified needs within the three months after the referral was made.

Data abstraction from VA chart review was the primary source of data collection. The methodology of the data abstraction was validated with providers of care coordination teams. Guidelines were established for consistency between the abstractors. A random sample of 10% of cases underwent a second independent abstraction by another trained abstractor of the research team blinded to the results of the first abstraction. Abstractors met regularly and discussed areas of potential ambiguity to reduce variation in data collection.

### Statistical analysis

Data were analyzed in the statistical analysis package, SPSS 18.0. Chi-square analysis was used to evaluate the proportions in the demographic comparisons between the two cohorts; accepted and redirected referrals for gender, race/ethnicity, age groups, geography, veteran status, and referral source.

We examined the frequency distributions of specific rehabilitation needs in the redirected referrals. Within the redirected patients that had a specific need, we evaluated what proportion of those patients received services to address their needs. We focused on the redirected referrals since admitted referrals are offered all necessary services for rehabilitation. 

Further case reviews were conducted on those offered rehabilitation services but did not receive the services, to determine if there were barriers or obstacles to accessing care. These specific services were sleep hygiene, pain management, and vision.

## Results

### Comparison of accepted patients and redirected patients

In this study sample for the period examined, there were a total of 1,025 referrals made to VAPAHCS PSC. These comprised the cohort analyzed. Of those, 119 were duplicate referrals of patients that were referred more than once to a PSC program (11.6%), including 12 referral cases that were referred to multiple VAPAHCS PSC programs. There were 906 unique patients that comprised the cohort referred to VAPAHCS PSC; 813 patients (89.7%) were accepted into VAPAHCS PSC, and 93 (10.3%) were redirected.  

The results of the demographic comparison analysis are displayed in Table 1.

**Table 1:** Demographic Analysis of the Patients Referred to the VA Palo Alto Health Care System Polytrauma System of Care

	Accepted	Redirected	P-value
Total	813	93	
Gender			P=0.066
Male	759 (93.4%)	82 (88.2%)	
Female	54 (6.6%)	11 (11.8%)	
Race/Ethnicity			p=0.446
Caucasian	437 (53.8%)	46 (49.5%)	
Non-Caucasian	377 (46.4%)	47 (50.5%)	
African-American	50 (6.2%)	4 (4.3%)	
Latino/Hispanic	105 (12.9%)	7 (7.5%)	
Multiple Ethnicities	29 (3.6%)	0	
Native American/Pacific Islander	29 (3.6%)	4 (4.3%)	
Asian American	47 (5.8%)	5 (5.4%)	
Declined to State/Unknown	116 (14.3%)	27 (29.0%)	
Age			p<0.001
18-30 years old	479 (58.9%)	31 (33.3%)	
31-60 years old	278 (34.2%)	44 (47.3%)	
>60 years old	56 (6.9%)	18 (19.4%)	
Service Status			p=0.039
Active Duty	76 (9.3%)	15 (16.1%)	
Veteran	737 (90.7%)	78 (83.9%)	
Geography (Residence)			p<0.001
California/West Coast	656 (80.7%)	49 (52.7%)	
Outside West Coast	157 (19.3%)	44 (47.3%)	
Referral Source			p<0.001
VAPA HCS	752 (92.5%)	33 (35.5%)	
Another VA	12 (1.5%)	36 (38.7%)	
Community (non-VA)	8 (1.0%)	10 (10.8%)	
Military Treatment Facility	41 (5.0%)	14 (15.1%)	

There was no difference in race/ethnicity between redirected patients versus accepted patients (53.8% of redirected patients were Caucasian vs. 49.5% of accepted patients, p=0.446). There were four areas of significant difference: females, age, active duty and veteran status, and geographical location. 

Females:

There was a larger proportion of females in redirected patients than accepted patients, 11.8% of redirected patients were female (n=11) vs. 6.6% of accepted patients (n=54), p=0.066). Further sub-analysis was conducted on the female patients. The sub-analysis focused on the referral diagnosis provided. We found that a higher proportion (18%, two of 11 female referrals) of females that were redirected had a concomitant spinal cord injury (SCI) diagnosis compared to the females accepted (no female referrals accepted had SCI). Generally, patients with a concomitant SCI are redirected to Spinal Cord Injury (SCI) rehabilitation units, co-located in the same facility. Multiple other diagnoses were provided in the female redirected referrals: mental health problems (45%), musculoskeletal (36%), substance abuse (18%), and PTSD (9%). 

Age:

Redirected patients were older than accepted patients (19.4% of redirected patients were over age 60 vs. 6.9% of accepted patients, p<0.001).

Active Duty and Veteran Status:

There were more active duty in the redirected patients (16.1% of redirected patients were active duty vs. 9.3% of accepted patients, p=0.039).

Geography:

There were significantly more California/West Coast states represented in the acceptances than the redirected referrals (52.7% of redirected patients were from California/West Coast vs. 80.7% of accepted patients, p<0.001). Additionally, the accepted patients had a very different referral source pattern than the redirected referrals (p<0.001), with most of the accepted patients (92.5%) being internally referred from VAPAHCS.

### Identification of redirected referral needs and determination of services to meet needs

The rehabilitative service needs and whether services were received in the 93 redirected patients is presented in Table 2.

**Table 2:** Identification of the Rehabilitative Service Needs in 93 Redirected Patients and Services Received

Rehabilitative Service Need	Identified Needs		Services Received	
	Total n=93	(% Total)		(% Within Need)
Psychological support	50	(54%)	38	(76%)
Mobility/physical therapy	49	(53%)	39	(80%)
Communication/speech language services	37	(40%)	30	(81%)
Occupational therapy	35	(38%)	30	(86%)
Medication	30	(32%)	25	(83%)
Pain management	26	(28%)	18	(69%)
Diet/feeding, nutrition	15	(16%)	13	(87%)
Vision	15	(16%)	11	(73%)
Hearing	12	(13%)	10	(83%)
Sleep hygiene	9	(10%)	3	(33%)

The most commonly identified patient needs were psychological support (a rehabilitative service need identified in 54% of the redirected patients) and mobility/physical therapy (53%) followed by communication/speech and language services (40%). In those that needed psychological support, 76% of these patients received appropriate psychological services within the three months after the initial referral. At least eighty percent (80%) of the redirected patients that needed physical therapy and speech language services received these services through the VA. The most frequently met needs for the redirected patients within the VA system were nutrition/feeding (87% of patients in need received the services), occupational therapy (86%), medication (83%), and hearing (83%).  

Patients in need of services received services least often in sleep hygiene (33%), pain management (69%), and vision (73%). Further case reviews were conducted on these patients to identify if there barriers to addressing these needs or providing these services. 

Sleep Hygiene:

Three out of nine patients (33%) had an identified sleep hygiene need that did not receive services. In further examination of these cases, these patients had severe co-morbid conditions, such as PTSD, substance abuse concerns, and/or other mental health issues. In these cases, the patients’ sleep concerns may have been co-occurring manifestations of these conditions. It was unclear whether sleep hygiene was enfolded in their treatments and the sleep disturbances persisted. 

Vision:

There were three out of 15 patients (20%) that had an identified vision need but did not receive services. In further examination of these cases, all had acute severe co-morbidities of PTSD, substance abuse, and homelessness. These patients were offered services but due to the acuity of the co-morbidities, the treatment needs of the co-morbidities superseded their vision needs.    

## Discussion

The formation of the VA Polytrauma System of Care (PSC) was initially to provide comprehensive care to those that were severely injured in the military conflicts in Iraq and Afghanistan. In the current political and military landscape, we anticipate the wind down of the war and further fiscal constraints. Therefore, it is vital for the PSC to examine the stewardship of resources while continuing our mission to provide access and outstanding care to the veterans and service members that meet admission criteria to our comprehensive rehabilitative services. We have an intense interest in ensuring equitable and appropriate services to the patients that require the specialized program we offer, given the significance of polytrauma/TBI. To better understand the overall impact of our service to polytrauma/TBI patients, whether or not they are accepted into our PSC program, we assessed the validity of our referral decision-making into the PSC program and confirmed that the identified needs of the patient referrals were addressed even if redirected to another program.

The PSC referral decision-making team is comprised of a multi-disciplinary team of Physical Medicine and Rehabilitation physicians, nurses, and social workers who consider each patient referral based on identification of needs, availability, accessibility, and prioritization of the services. The decision-making process assesses the needs of the referred patients, decides whether the appropriate highly specialized clinical care offered at the VAPAHCS PSC would best meet the needs of the patient and their family, and balances this with availability of resources.          

Given the specificity and breadth of the comprehensive polytrauma resources, it is important our decisions are valid, and the patients in need of such specialized care are receiving them. On the West Coast, the reach of the PSC is broad under a “hub and spoke” system, where smaller VA facilities (“spokes”) located within the Veterans Integrated Service Network Sierra Pacific (VISN 21) which includes parts of Nevada, central/northern California, Hawaii, and the vast Pacific Rim, can consult with the major “hub” facility located in Palo Alto. It was important for the PSC that all referrals independent of acceptance were provided services, if not within the PSC but within the VA system. 

Referral decisions are based on administrative eligibility, and ability to participate in treatments. Patients are accepted into the PSC for evaluation and comprehensive treatment. Accepted patients meet admission criteria, which includes that at admission the patient would be appropriate for evaluation and/or rehabilitative care. After a patient is accepted and later admitted into the PSC, they are offered all the necessary evaluation and rehabilitative services to assist in the attainment of the patients’ and institutional goals. 

We objectively assessed the demographic characteristics of patients accepted into the VAPAHCS PSC compared to patients who were redirected to ensure that we were without bias in our decision-making. We found that the identified differences were appropriate and even further examined as a foundation for future studies. Significant differences were noted in gender, military status, geography, and age. These differences prompted more in-depth assessment to evaluate for bias. 

*Gender distribution *differed between the accepted and redirected referrals. Females were more likely to be redirected. Of those that were redirected, 11.8% were female, compared to those accepted (6.6% female). Therefore, we further conducted a subgroup analysis examining the female referral population. This revealed that more females had a concomitant spinal cord injury (SCI), and therefore, they were more appropriately referred to an SCI program for further examination. However, this examination revealed that females represent a low percentage of all referrals made to the VAPAHCS PSC (7%) and raises concerns that any gender-related differences may be overlooked when examining the larger predominantly male referral group. Using more specific methodologies, this has provided preliminary foundational work for further research on gender-related outcome differences in the polytrauma/TBI populations. 

*Geography* was also an area that yielded significant differences between the accepted and redirected referrals. We found that the patients accepted into the VAPAHCS PSC were more likely to have permanent residence in California or the West Coast. This finding is not surprising given a closer geographic proximity of a referral source to the VA Palo Alto Health Care System will generally lead to referral sources that are better informed of the PSC service offerings.  Generally, better informed referral sources lead to more appropriate referrals made to the PSC.  Furthermore, the services will be more accessible to the patients, especially given the far-reaching distribution of the Polytrauma Support Care Teams (PSCT) located nationwide throughout VA medical facilities. Similarly, referrals are often made from referral sources with consideration of the family and caregiver preferences and anticipated disposition of the patient. This is reflective of the goals of geomapping.      

*Military status* also differed significantly between the accepted and redirected referrals.  Whereas about 9% of those accepted into the PSC were active duty during the time of their referral, a slightly higher percentage of those redirected were active duty (16%). The active duty service member population has access to other military facilities and services that also provide specialized care and treatment. The decision-making around the active duty service member takes into consideration other potential issues, such as military command. 

*Age* differed significantly between the accepted and redirected referrals. The accepted patients tended to be younger than those that were redirected. Two-thirds of the cohort of older patients (age >60) were redirected to seek a lower level of care, such as general comprehensive rehabilitation, than what the specialized polytrauma/TBI care offers.

We concluded that the PSC acceptance decisions were effective and appropriate, and were without selection bias. The process review has allowed more focused education efforts to inform referral sources of the admission criteria and to ensure that all referred patients are definitively receiving the appropriate level of care. The education efforts have included visits to referral sites, including military treatment facilities, developing publication materials, and addressing inquiries.

This study is unique in that we did not solely focus on the accepted referrals, but we also examined whether the needs of the redirected patients were also being met. The methodology used to assess the referrals was feasible because the national computerized patient record system (CPRS) that allows a comprehensive review of the records of the PSC cohort. Recent integration of medical records between the Department of Defense and the VA strengthened our ability to follow patients and assess the care received, be it at VAPAHCS, another VA hospital, or military treatment facility (MTF).   

We found that the patients who were redirected had the majority of their needs met within three months after their referral with services they received primarily through other VA agencies (Table 2). The top rehabilitation needs in redirected patients were psychological support, mobility/physical therapy, and communication/speech language services. These needs were often met at another VA setting. This indicates that the VA system, including the VAPAHCS PSC, functions to provide identified needed services to the VA population. In all cases, services were not received because the patient was being treated for more acute conditions within the time frame we reviewed. 

### Study limitations

Not all patients utilize health care services solely through the VA or other military treatment facilities. In some cases, patients who are accepted into the PSC decide to find care elsewhere, withdraw, and are not admitted into the PSC. Therefore, the data presented may be an underestimate of the overall health services utilized since patients may have found alternative care through other health service organizations. 

This review was limited to the referral data of the VAPAHCS PSC. Nationally, to be eligible for admission into the VA PSC, the patient must be: 

1) a medically stable veteran and service member, 

2) have sustained multiple physical, cognitive, and/or emotional injuries secondary to trauma, 

3) not require one-to-one staffing for medical or behavioral reasons, 

4) not require a ventilator to breathe, and 

5) have potential to benefit from rehabilitation or need an initial, comprehensive rehabilitation evaluation and care plan.          

We acknowledge the limitation that even when a patient is offered and has received services that this may not be enough to resolve the actual patient need. However, if a patient IS offered and receives services, it is an indication that the health care system is effective in ensuring that services are available to address the patient’s needs. However, we conclude from this review that most patients referred to the VAPAHCS are offered and received needed services. This includes those patients accepted into the PSC system who received all PSC services, and those that were redirected. 

## Conclusions

The acceptance decisions for referrals made to the VAPAHCS PSC were appropriate and without bias, and effective in ensuring access to the specialized and comprehensive services that the PSC offers to polytrauma patients. Patients that were redirected had the majority of their needs met with the services they received through other VA agencies. 
